# Modulation of myocardial contraction by peroxynitrite

**DOI:** 10.3389/fphys.2012.00468

**Published:** 2012-12-12

**Authors:** Mark J. Kohr, Steve R. Roof, Jay L. Zweier, Mark T. Ziolo

**Affiliations:** ^1^Department of Physiology and Cell Biology, Davis Heart and Lung Research Institute, The Ohio State UniversityColumbus, OH, USA; ^2^Division of Cardiovascular Pathology, Department of Pathology, Johns Hopkins UniversityBaltimore, MD, USA; ^3^Department of Internal Medicine: Division of Cardiovascular Medicine, Davis Heart and Lung Research Institute, The Ohio State UniversityColumbus, OH, USA

**Keywords:** myocyte, peroxynitrite, nitric oxide synthase, Ca^2+^, cAMP-dependent protein kinase, protein phosphatase 2a

## Abstract

Peroxynitrite is a potent oxidant that is quickly emerging as a crucial modulator of myocardial function. This review will focus on the regulation of myocardial contraction by peroxynitrite during health and disease, with a specific emphasis on cardiomyocyte Ca^2+^ handling, proposed signaling pathways, and protein end-targets.

The heart plays a vital role in the cardiovascular system by providing the specialized tissues and organs of the body with a continual supply of oxygen and other essential nutrients. Modulation of myocardial contraction allows the heart to meet the demands of the body despite continual changes in metabolism. This modulation occurs at several different levels, including the level of the ventricular cardiomyocyte.

## Excitation–contraction coupling

Cardiomyocyte contraction occurs via a process termed excitation–contraction coupling, in which the electrical activity of the heart is translated into cardiomyocyte contraction (Bers, 2001, 2002). This process commences with the cardiac action potential and the depolarization of the myocyte membrane, which leads to the opening of L-type Ca^2+^ channels (Bers, 2001, 2002). This causes an influx of Ca^2+^ into the myocyte via the L-type Ca^2+^ current (I_Ca_), and subsequently triggers the opening of the sarcoplasmic reticulum (SR) Ca^2+^-release channels or ryanodine receptors (RyRs). This results in an efflux of Ca^2+^ from the SR, which is the major Ca^2+^ store in the cardiomyocyte, in a process termed Ca^2+^-induced Ca^2+^-release. This process initiates the upstroke of the myocyte [Ca^2+^]_i_ transient and this Ca^2+^ is now available for myocyte contraction. SR Ca^2+^ load, which represents the Ca^2+^ available for release from the SR, is a critical determinant of [Ca^2+^]_i_ transient amplitude and thus myocyte contraction (Bassani et al., 1995; Trafford et al., 1995).

The [Ca^2+^]_i_ transient is responsible for the activation of cardiomyocyte contraction or shortening. When [Ca^2+^]_i_ increases within the cardiomyocyte, this Ca^2+^ diffuses to the myofilaments and binds to troponin C (TnC) (Davis and Tikunova, 2008). This induces a strong interaction between TnC and troponin I (TnI), effectively destabilizing the interaction between TnI and actin. This shifts the troponin–tropomyosin complex in such a way as to allow the myosin head to bind directly to actin, thus leading to the formation of a myosin crossbridge. Provided that ATP is readily available to the myosin head, myocyte force production and/or shortening ensues until Ca^2+^ is no longer bound to TnC.

Relaxation is initiated by the decline of the [Ca^2+^]_i_ transient, which is primarily mediated by the SR Ca^2+^-ATPase/phospholamban complex (SERCA/PLB) and the Na^+^/Ca^2+^ exchanger (NCX) (Bers, 2001, 2002). The SERCA/PLB complex serves to re-sequester Ca^2+^ into the SR, while NCX directly extrudes Ca^2+^ into the extracellular space. PLB is a phosphoprotein that plays a critical role in the decline of the [Ca^2+^]_i_ transient by regulating the uptake of Ca^2+^ by SERCA. Under basal conditions, PLB remains hypophosphorylated and serves to inhibit the uptake of Ca^2+^ by SERCA. This inhibition can be relieved upon PLB Serine16 phosphorylation by protein kinase A (PKA), thus allowing greater uptake of Ca^2+^ into the SR (Chu et al., 2000; Hagemann and Xiao, 2002; Mattiazzi et al., 2006). There are two main serine/threonine protein phosphatases that dephosphorylate PLB, protein phosphatase 1 (PP1) and protein phosphatase 2a (PP2a) (Macdougall et al., 1991).

## Modulation of excitation–contraction coupling

Since every cardiomyocyte is recruited to contract with each heartbeat, numerous endogenous systems have evolved to effectively regulate the ventricular myocyte contractile state by modulating excitation–contraction coupling. For example, stimulation of the β-adrenergic receptor (β-AR) pathway results in positive inotropic and lusitropic effects via modulation of key excitation–contraction coupling proteins (Bers and Ziolo, 2001). Activation of the β-AR signaling pathway transpires with binding of an agonist (e.g., epinephrine, isoproterenol) to the receptor, which results in the dissociation of the G_sα_ subunit. G_sα_ induces cAMP production via adenylate cyclase (AC). Increased cAMP then activates the cAMP-dependent protein kinase or PKA, which phosphorylates many different targets in the cardiomyocyte, including I_Ca_, RyR, TnI, and PLB. The end result is increased Ca^2+^ influx, increased SR Ca^2+^ uptake, load, and release, and decreased myofilament Ca^2+^ sensitivity. PLB phosphorylation at Serine16 by PKA is one of the primary mechanisms responsible for the observed positive inotropic and lusitropic effects of β-AR stimulation (Chu et al., 2000; Li et al., 2000; Roof et al., 2011, 2012). Therefore, altering PLB phosphorylation levels will lead to drastic changes in myocardial contraction and relaxation. Since the β-AR pathway is perhaps the most important regulator of myocardial contractility, this pathway itself is modulated by other endogenous systems such as reactive nitrogen species (RNS).

## Reactive nitrogen species

RNS are quickly emerging as crucial modulators of myocardial contraction during health and disease (Pacher et al., 2007). Alterations in the production of these reactive species may be responsible for part of the dysfunction that is observed during many pathophysiological conditions of the myocardium and may represent novel therapeutic targets. However, the modulation of myocardial function by RNS is a relatively new field and many questions remain unanswered. For example, studies have yet to conclusively determine whether this dysfunction results from the loss of endogenous RNS production [i.e., nitric oxide (NO)] or from the production of supraphysiological levels of reactive species. In all likelihood, this dysfunction stems from the partial loss of localized RNS signaling coupled with an increase in the production of more reactive species, including peroxynitrite. Indeed, this appears to be the case (Feldman et al., 2008; Ziolo et al., 2008).

NO is a well-known modulator of many physiological processes, including neural transmission and the regulation of blood pressure. However, NO is also surfacing as a key modulator of myocardial function by serving as a regulator of excitation–contraction coupling (Ziolo et al., 2001b, 2004; Wang et al., 2008a,b, 2009, 2010) and β-AR signaling (Balligand et al., 1993; Ziolo et al., 2001c). NO is produced endogenously within the myocardium by three distinct isoforms of NO synthase (NOS) (Ziolo and Bers, 2003). Neuronal NOS (NOS1) and endothelial NOS (NOS3) are constitutively expressed within cardiomyocytes. NOS1 and NOS3 produce low amounts of NO in phase with the myocyte [Ca^2+^]_i_ transient. Inducible NOS (NOS2), on the other hand, is only expressed within cardiomyocytes during inflammatory responses which occur during many pathophysiological conditions of the myocardium. NOS2 produces much higher amounts of NO compared to constitutive isoforms and is [Ca^2+^]_i_ independent (Schulz et al., 1992; Ziolo et al., 1998, 2001a).

NO is known to signal through at least two distinct signaling pathways: cGMP-dependent and cGMP-independent (Ziolo, 2008). cGMP-dependent signaling occurs through the activation of guanylate cyclase and protein kinase G (PKG), while cGMP-independent signaling primarily occurs via direct protein modification (e.g., *S*-nitrosylation) (Martinez-Ruiz and Lamas, 2004; Hess et al., 2005; Handy and Loscalzo, 2006; Kohr et al., 2011b). NO has also been shown to directly activate AC independent of β-AR activation, thus resulting in positive inotropic effects via increased cAMP production and PKA activation (Vila-Petroff et al., 1999). NO also signals by coupling to other reactive signaling species (e.g., superoxide anion) to form related congeners, such as peroxynitrite.

## Peroxynitrite

Peroxynitrite (ONOO^−^) is a potent oxidant that is formed upon the reaction of NO and superoxide. This reaction occurs with an extremely high rate constant, making the formation of peroxynitrite quite favorable (Huie and Padmaja, 1993; Beckman and Koppenol, 1996). Although peroxynitrite is a highly reactive species, it is still capable of diffusing through biological membranes and interacting with intracellular constituents (Denicola et al., 1998). Under physiological conditions, peroxynitrite production remains low and potential oxidative damage is reduced via endogenous cellular antioxidant defenses (Radi et al., 2002a,b). Low levels of peroxynitrite may also serve to modulate various intracellular signaling pathways. In fact, peroxynitrite appears to play a role in the regulation of many important physiological processes. For example, peroxynitrite has been demonstrated to modulate T cell proliferation (Brito et al., 1999; Vig et al., 2004), platelet aggregation (Schildknecht et al., 2008), and neutrophil adhesion (Zouki et al., 2001). Peroxynitrite is thought to mediate these physiological processes by targeting various proteins and/or signaling pathways, including receptor tyrosine kinases, mitogen-activated protein kinases, phosphoinositide 3-kinase/protein kinase B (Akt), protein kinase C, and nuclear factor κB (Pacher et al., 2007).

The production of peroxynitrite increases greatly during the pathogenesis of numerous disorders, such as cerebral ischemia-reperfusion (Thiyagarajan et al., 2004; Dhar et al., 2006), myocardial ischemia-reperfusion (Wang and Zweier, 1996; Cheung et al., 2000), heart failure (Ferdinandy et al., 2000; Mihm et al., 2001; Zhang et al., 2007), atherosclerosis (Buttery et al., 1996; Luoma et al., 1998), diabetes (Suarez-Pinzon et al., 1997, 2001), and septicemia (Bhattacharyya et al., 2004). Although peroxynitrite production plays a critical role for neutrophils and macrophages in microbial defense (Macmicking et al., 1997; Nathan and Shiloh, 2000), supraphysiological levels of peroxynitrite are almost always detrimental to cellular function. Peroxynitrite exerts damaging effects by altering protein structure and function via irreversible nitration to tyrosine residues and cysteine oxidation (Kamat, 2006; Pacher et al., 2007). Peroxynitrite can also exert effects by reacting with transition metal centers, including heme prosthetic groups (Pietraforte et al., 2001; Lin et al., 2007) and zinc-thiolate motifs (Crow et al., 1995; Zou et al., 2004), thus resulting in the inactivation of many enzymes. NOS3, an enzyme which exerts many cardioprotective effects (Janssens et al., 2004; Buys et al., 2007; Wang et al., 2008b, 2012), can be inhibited upon exposure to high levels of peroxynitrite (Zou et al., 2004; Chen et al., 2010). Additionally, hydroxylation of ring systems (Szabo et al., 1997; Tuo et al., 1998), DNA strand breakage (Szabo et al., 1996, 1997), and lipid peroxidation (Radi et al., 1991a,b) can be induced by peroxynitrite. Apoptosis and necrosis can also be triggered upon peroxynitrite exposure (Gilad et al., 1997; Arstall et al., 1999; Pacher et al., 2002; Levrand et al., 2006). Moreover, peroxynitrite will react with other molecules, such as carbon dioxide (CO_2_), to form additional reactive species (i.e., nitrosoperoxycarbonate) (Uppu and Pryor, 1996). Increased peroxynitrite production is further compounded by the depletion of endogenous antioxidant pools, including glutathione (GSH), which is a major scavenger of reactive oxygen and nitrogen species (Valko et al., 2007). The production of peroxynitrite occurs endogenously in several different ways.

Peroxynitrite can be formed endogenously within the ventricular cardiomyocyte through various routes under physiological and pathophysiological conditions. A major source for the production of peroxynitrite includes the NOS isoforms (namely NOS1 and NOS2) and via non-classical pathways (namely nitroxyl).

NOS1 has been shown to co-immunoprecipitate with xanthine oxidoreductase (Khan et al., 2004), a superoxide producing enzyme. This interaction between NOS1 and xanthine oxidoreductase will lead to the physiological production of low levels of peroxynitrite. Additionally, NOS1 can potentially produce both NO and superoxide (Pou et al., 1992; Xia et al., 1996), although this is more likely to occur with NOS uncoupling during various disease states (Sun et al., 2008). NOS uncoupling will increase superoxide production by NOS1, while decreasing NO production. NOS1 is considered to be a physiological regulator of myocardial function by increasing basal contractility, the force-frequency response, and the response to β-AR stimulation (Barouch et al., 2002; Khan et al., 2003; Vandsburger et al., 2007; Wang et al., 2008a, 2010).

NOS2 is a high output NOS isoform and can readily become uncoupled upon depletion of the cofactors necessary for NO production, thus leading to the production of both NO and superoxide (Xia and Zweier, 1997; Mungrue et al., 2002). Additionally, NADPH oxidase and xanthine oxidoreductase can increase superoxide production under the same pathophysiological conditions in which NOS2 is expressed (Heymes et al., 2003; Minhas et al., 2006). This will lead to the production of supraphysiological levels of peroxynitrite. NOS2 is considered to be a pathophysiological regulator of myocardial function by decreasing the response to β-AR stimulation (Drexler et al., 1998; Ziolo et al., 2001b, 2004).

In addition to the NO produced via NOS1 and NOS2, additional sources of peroxynitrite include nitroxyl, the one electron reduction production of NO (Kirsch and De Groot, 2002). Endogenous production of nitroxyl within cardiomyocytes may occur through various routes, including via hydrogen atom extraction by NO (Akhtar et al., 1985). NOS1 also remains a potential source for the production of nitroxyl (Schmidt et al., 1996; Ishimura et al., 2005). Therefore, the reaction of nitroxyl with O_2_ will lead to the production of low levels of peroxynitrite.

## Peroxynitrite and cardiomyocyte contraction

Recent work has now demonstrated that peroxynitrite is able to modulate excitation–contraction coupling, and hence contractility. In the myocardium, peroxynitrite has been demonstrated to exert biphasic effects on cardiomyocyte contraction. More specifically, low levels of peroxynitrite (which occurs under physiological conditions) have been shown to produce positive inotropic effects on cardiomyocyte function. Conversely, high levels of peroxynitrite (which occurs under pathophysiological conditions) are extremely detrimental to myocardial function by decreasing cardiomyocyte contraction.

## Physiological regulation of cardiomyocyte contraction by peroxynitrite

Many studies have shown that low levels of peroxynitrite induce positive inotropic effects on basal myocardial function. Chesnais et al. demonstrated peroxynitrite to increase force production in frog atrial and ventricular fibers using authentic peroxynitrite, as well as the peroxynitrite donor SIN-1 (Chesnais et al., 1999a,b). Paolocci et al. confirmed this positive effect on basal function in mammalian *ex vivo* myocardial preparations using SIN-1 (Paolocci et al., 2000). Interestingly, this effect was reversed with GSH and occurred independently from global changes in cAMP and cGMP levels. Furthermore, we demonstrated that a low concentration of peroxynitrite increased basal and submaximal β-AR-stimulated contraction in isolated cardiomyocytes (Kohr et al., 2008a). We next investigated the molecular mechanisms of this peroxynitrite-induced increase in basal contraction and found that it was dependent upon PLB. That is, peroxynitrite had no effect on basal contraction in PLB knockout myocytes. Further explorations demonstrated that peroxynitrite resulted in the enhancement of PLB Serine16 (the PKA site) phosphorylation (Kohr et al., 2010) and was abolished upon inhibition of PKA with KT5720 (Kohr et al., 2010). Low peroxynitrite also increased PKA activity in cardiac homogenates and in purified preparations of PKA containing both the regulatory and catalytic subunits of PKA, indicating that peroxynitrite induces a direct, cAMP-independent activation of PKA. This direct effect may occur via *S*-nitrosylation, *S*-glutathiolation and/or cysteine oxidation (Ferdinandy, 2006; Pacher et al., 2007), as PKA has several cysteine residues which are susceptible to these types of modifications (Brennan et al., 2006; Burgoyne and Eaton, 2009; Kohr et al., 2011a). Thus, low levels of peroxynitrite shift the kinase/phosphatase balance leading to increased PLB phosphorylation. Interestingly, the effect of low peroxynitrite on submaximal β-AR-stimulated contraction was still observed in PLB knockout myocytes, suggesting another protein target. A recent study demonstrated that PKA phosphorylation of RyR contributes to the inotropic effect of β-AR stimulation using a low dose of isoproterenol (Shan et al., 2010). Thus, peroxynitrite-mediated activation of PKA may also lead to RyR phosphorylation contributing to the contractile effects of β-AR stimulation. Non PKA-mediated effects of peroxynitrite have also been reported such as *S*-nitrosylation of SERCA2a, which increases Ca^2+^ uptake and relaxation (Bencsik et al., 2008).

We previously demonstrated in healthy ventricular cardiomyocytes that NOS1 signals partly through the production of peroxynitrite (Wang et al., 2008a). Additionally, we and others have shown that NOS1 signaling increases myocardial contraction (basal, force-frequency response and β-AR-stimulated contraction) (Barouch et al., 2002; Khan et al., 2004; Wang et al., 2008a), parameters that can be considered physiologic. These effects are consistent with the positive inotropic effects of peroxynitrite (i.e., peroxynitrite increased basal and submaximal β-AR-stimulated contraction). Interestingly, NOS1^−/−^ myocytes exhibit reduced basal contraction and a diminished response to β-AR stimulation compared to wild type (Barouch et al., 2002; Khan et al., 2004; Wang et al., 2008a), which we and others demonstrated to occur in part through a reduction in RyR activity (Wang et al., 2010) and PLB Serine16 phosphorylation (Wang et al., 2008a; Zhang et al., 2008). This is also consistent with the PLB and RyR dependent effects of low peroxynitrite (Kohr et al., 2008a). In addition, NOS1 has been shown to potentially modulate PKA activity, as PKA inhibition decreased PLB Serine16 phosphorylation and the rate of relaxation in wild-type myocytes, but had no effect in NOS1^−/−^ myocytes (Zhang et al., 2008). Therefore, the physiological production of low levels of peroxynitrite may serve to maintain and/or increase basal and β-AR-stimulated myocyte contraction, in part, through a direct, cAMP-independent activation of PKA. Further, the loss of localized NOS1-mediated peroxynitrite production under certain disease states where NOS1 translocates to the sarcolemma (Damy et al., 2003, 2004) may result in decreased myocyte contraction similar to that observed with the knockout of NOS1.

## Pathophysiological regulation of cardiomyocyte contraction by peroxynitrite

At high concentrations, peroxynitrite is detrimental to myocardial function. Studies examining high levels of peroxynitrite have demonstrated dysfunctional effects via direct exposure or using peroxynitrite donors (Lopez et al., 1997; Ma et al., 1997; Ferdinandy et al., 1999, 2000; Katori et al., 2006). Additionally, we have demonstrated a NOS2-induced reduction in β-AR-stimulated RyR activity through a cGMP-independent mechanism, likely via peroxynitrite (Ziolo et al., 2001b). Further, we and others have shown a reduction in β-AR-stimulated contraction upon exposure to a high concentration of SIN-1 in isolated cardiomyocytes (Stojanovic et al., 2001; Yin et al., 2002). We demonstrated that this SIN-1-induced decrease in β-AR-stimulated contraction occurred via peroxynitrite formation and was also dependent upon PLB. However, unlike low peroxynitrite, high peroxynitrite results in a reduction in PKA-dependent PLB Serine16 phosphorylation (Kohr et al., 2008b). Upon further examination of this signaling pathway, we determined that high peroxynitrite increased phosphatase activity in cardiac homogenates (low peroxynitrite had no effect on phosphatase activity). Interestingly, it appears that peroxynitrite selectively targets PP2a as we observed an increased interaction between PLB and PP2a (but not PP1) with peroxynitrite (Kohr et al., 2009). This reversible effect may occur through a protein intermediate or from the direct modification of PP2a by peroxynitrite via *S*-nitrosylation, *S*-glutathiolation, and/or cysteine oxidation (Ferdinandy, 2006; Pacher et al., 2007). PP2a has several cysteine residues which are susceptible to these types of modifications (Sommer et al., 2002). Consistent with our results, peroxynitrite has been shown to directly activate PP2a via tyrosine nitration in endothelial cells (Wu and Wilson, 2009). Thus, high levels of peroxynitrite shift the kinase/phosphatase balance leading to decreased PLB phosphorylation. While PP2A also modulates RyR phosphorylation (Terentyev et al., 2003), we did not observe any effects of high peroxynitrite on the β-AR response in PLB knockout myocytes, which suggests that this may be a compartmentalized effect targeted to PLB.

The production of high levels of peroxynitrite in the myocardium is often associated with the expression of NOS2 (Xia et al., 1998; Mungrue et al., 2002) and increased ROS production via NADPH oxidase (Byrne et al., 2003; Heymes et al., 2003) and xanthine oxidase (Tziomalos and Hare, 2009). Specifically in heart failure, studies have shown an increase in peroxynitrite production (Ferdinandy et al., 2000; Mihm et al., 2001; Zhang et al., 2007), as well as a diminished response to β-AR stimulation (Houser et al., 2000). We have previously demonstrated that myocytes isolated from failing human hearts expressing NOS2, displayed a blunted response to β-AR stimulation (Ziolo et al., 2004). This dysfunction could be reversed upon acute inhibition of NOS2, such that peak [Ca^2+^]_i_ and cell shortening were significantly increased. This same functional phenomenon was observed with high peroxynitrite (i.e., peroxynitrite decreased contraction during β-AR stimulation). Interestingly, PP2a activity has been shown to be increased in heart failure (Boknik et al., 2000), while PLB Serine16 phosphorylation has been shown to be decreased (Bartel et al., 1996; Schwinger et al., 1999; Sande et al., 2002). Therefore, the increased formation of peroxynitrite that occurs in heart failure may be an important component of the β-AR dysfunction observed in heart failure and other cardiomyopathies. A recent study demonstrated that peroxynitrite was an independent risk predictor of post-operative complications (i.e., atrial fibrillation, need for inotropic support, length of hospital stay) among patients undergoing cardiac surgery for various reasons (Antoniades et al., 2012), further validating the significance of increased peroxynitrite in cardiac dysfunction.

## Peroxynitrite and mitochondrial function

The processes of cardiomyocyte contraction and Ca^2+^ reuptake/extrusion are dependent upon ATP. Through oxidative phosphorylation, mitochondria produce the majority of the ATP that is necessary for these processes. Ca^2+^ is also taken up by the mitochondria during each Ca^2+^ transient, and this serves to couple excitation–contraction coupling with energetic demand (Maack and O'Rourke, 2007). Recent work has demonstrated that mitochondrial function is modulated by RNS (i.e., NO, peroxynitrite).

NO is a well-established physiological mediator of mitochondrial respiration, and these effects primarily result from the inhibition of Complex I and IV of the electron transport chain (Brookes, 2004). Although Complex I and IV are similarly inhibited by NO, this inhibition occurs through two distinct mechanisms. In the case of Complex I, NO acts via *S*-nitrosylation of the 75 kDa subunit of Complex I (Burwell et al., 2006). Complex IV, on the other hand, is inhibited through the reaction of NO with the catalytic metals located in the active site of cytochrome *c* oxidase (Cleeter et al., 1994; Palacios-Callender et al., 2004). NO can also target additional mitochondrial proteins by reacting with superoxide to yield peroxynitrite. The effects of peroxynitrite on mitochondrial function are much less defined when compared to those of NO, but peroxynitrite has the potential to signal physiologically in the mitochondria or to contribute to mitochondrial dysfunction during pathological states.

Peroxynitrite has been shown to target many different proteins in the mitochondria and this can occur via *S*-nitrosylation, as reported to occur with Complex I of the electron transport chain (Borutaite et al., 2000), or through irreversible nitration and/or cysteine oxidation. The reversibility of *S*-nitrosylation may represent a potential physiological signaling pathway for peroxynitrite in the mitochondria, while the effects of irreversible nitration and cysteine oxidation are likely to be highly detrimental to mitochondrial function. For example, peroxynitrite induced the irreversible inhibition of mitochondrial creatine kinase, and this effect could not be reversed with GSH treatment (Konorev et al., 1998). Peroxynitrite was also shown to inactivate the Kreb's cycle enzyme aconitase (Castro et al., 1994). Additional mitochondrial targets of peroxynitrite include the adenine nucleotide translocase (Vieira et al., 2001), nicotinamide nucleotide transhydrogenase (Forsmark-Andree et al., 1996), and Complex I, II, and V of the electron transport chain (Radi et al., 1994; Cassina and Radi, 1996; Riobo et al., 2001). Manganese superoxide dismutase, which is a major enzyme for the decomposition of superoxide in the mitochondria, is another target of peroxynitrite and is inactivated via nitration (Quijano et al., 2001). This peroxynitrite-induced inactivation is further compounded by increased levels of superoxide, which could in turn lead to the formation of additional peroxynitrite. Hence, a decrease in energy supply by inhibiting mitochondrial function could contribute to the depressed contractile function associated with high levels of peroxynitrite.

## Low vs. high levels of peroxynitrite

Peroxynitrite has a biphasic effect on cardiomyocyte contraction, which is mainly concentration dependent (low vs. high). However, since peroxynitrite is highly reactive and rapidly decomposed, accurate measurements of concentration are difficult. In addition, determining intracellular concentrations of peroxynitrite with exogenously applied authentic peroxynitrite or the peroxynitrite donor SIN-1 is challenging, making direct comparisons to endogenously produced peroxynitrite formidable. In general, concentrations of 30 μM or less of authentic peroxynitrite or 100 μM or less of SIN-1 will result in positive inotropic effects as described. We have previously reported that 10 μM SIN-1 produces 3 nM/min peroxynitrite in our physiological saline solution (Kohr et al., 2008a). Higher concentrations (100 μM authentic peroxynitrite or 200 μM SIN-1) will result in negative inotropic effects as described. We have previously reported that 200 μM SIN-1 results in a 6X greater production of peroxynitrite compared to 10 μM SIN-1 (Kohr et al., 2008b). We consider these levels to be physiologically and pathophysiologically relevant as we and others observed similar effects on function in studies investigating NOS1 and NOS2 signaling. Thus, these concentrations of peroxynitrite result in the activation of various signaling pathways (PKA, PP2A, S-nitrosylation, etc.) resulting in the enhancement or reduction of myocardial contractility. There have been exceptions reported in which low concentrations of authentic peroxynitrite/SIN-1 resulted in negative inotropic effects or high concentrations of SIN-1 resulted in positive inotropic effects (Yin et al., 2002; Katori et al., 2006; Kohr et al., 2008a). Thus, other factors also contribute to the contractile effects of peroxynitrite such as cardiomyocyte contractile state, signaling pathway activation (e.g., *S*-nitrosylation, nitration, cGMP or cAMP) as well as the length of exposure (Schulz et al., 1997; Ziolo, 2008).

Studies have also been performed using very high concentrations of authentic peroxynitrite (mM) or long exposure times. These studies revealed irreversible effects of peroxynitrite that depressed myocyte contraction and inactivated SERCA (Ishida et al., 1996; Knyushko et al., 2005; Lokuta et al., 2005). A consequence of this very high concentration of peroxynitrite is likely a toxic effect that results in cell damage. For example, peroxynitrite is able to activate matrix metalloproteinases that will result in the cleavage of α-actinin and TnI (Wang et al., 2002; Rork et al., 2006; Sung et al., 2007; Leon et al., 2008). We believe that very high concentrations of peroxynitrite can be produced in certain disease states such as heart failure or ischemia/reperfusion injury which have reported SERCA inactivation via nitration or TnI cleavage.

**Figure 1:**
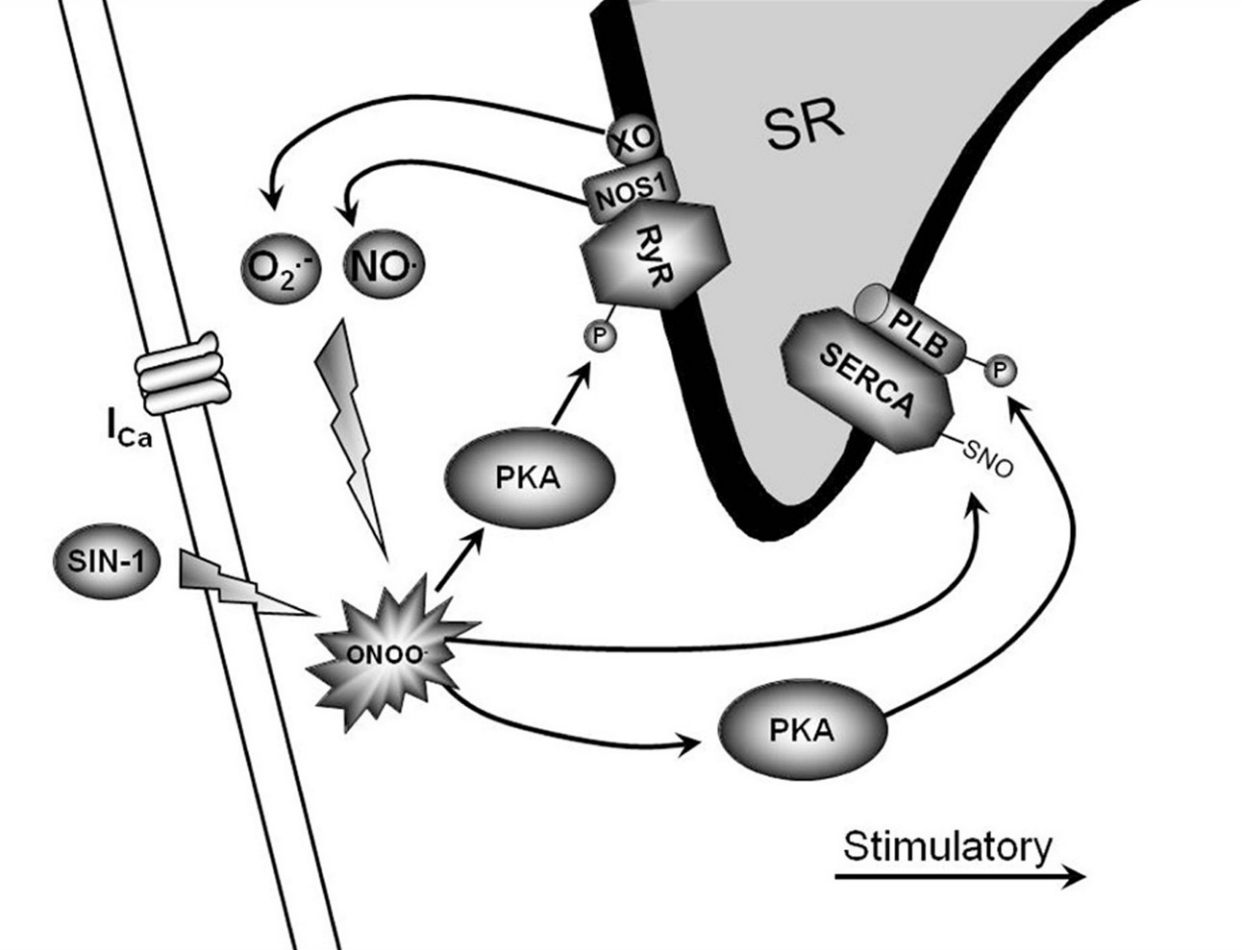
**Physiological signaling pathway of low peroxynitrite.** This is a diagrammatic representation of low peroxynitrite signaling and various proposed end targets within the cardiomyocyte. The endogenous production of low peroxynitrite can occur via NOS1/xanthine oxidase.

**Figure 2:**
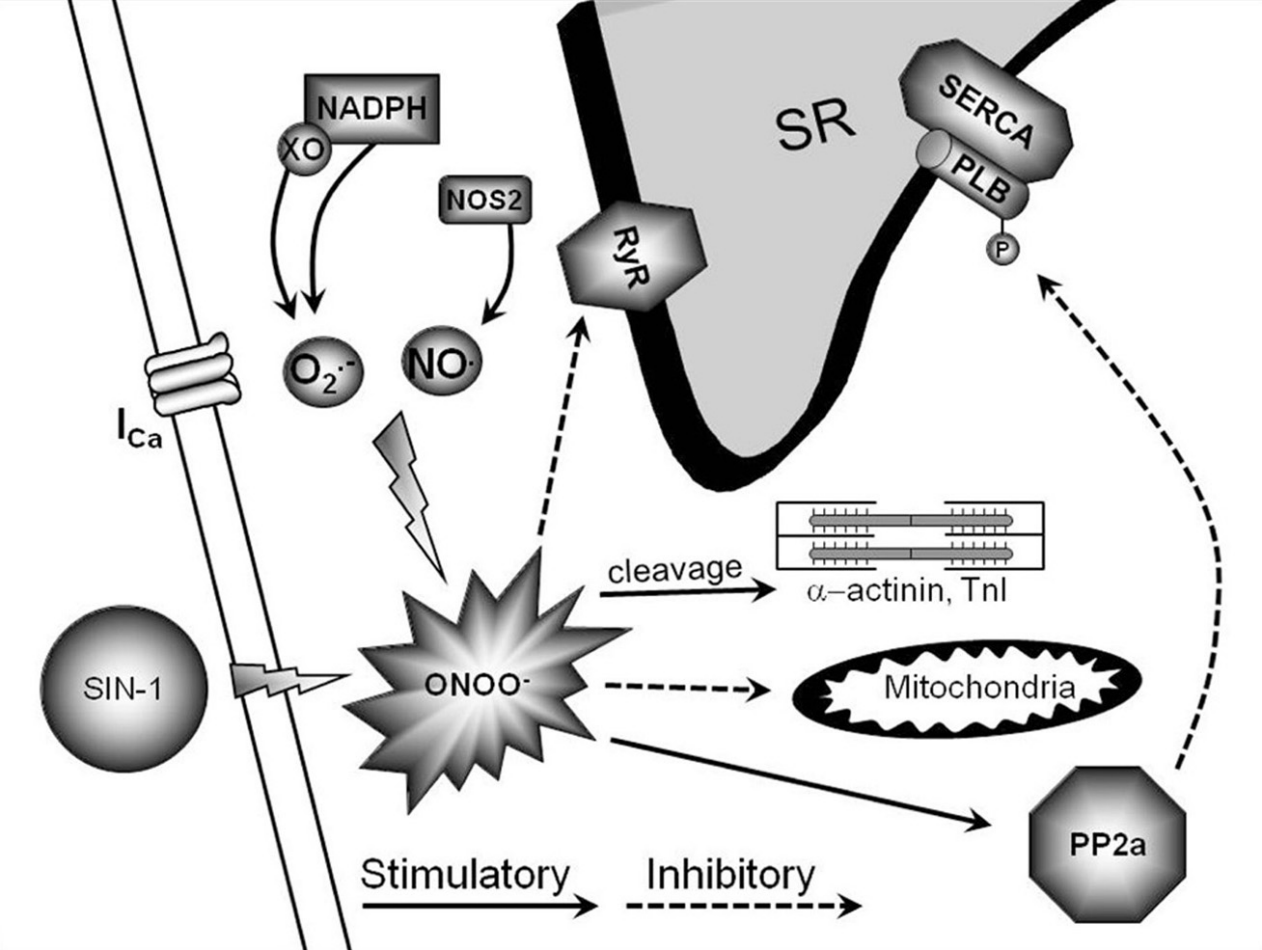
**Pathophysiological signaling pathway of high peroxynitrite.** This is a diagrammatic representation of high peroxynitrite signaling and various proposed end targets within the cardiomyocyte. The endogenous production of high levels of peroxynitrite can occur via NOS2/NADPH oxidase/xanthine oxidase.

In conclusion, peroxynitrite is emerging as a crucial modulator of myocardial function during health and disease. Under physiological conditions, low levels of peroxynitrite serve to maintain and/or increase basal and β-AR-stimulated contraction in the myocardium. This occurs via RyR and PLB phosphorylation through direct PKA activation and SERCA activation via S-nitrosylation (Figure 1). Conversely, supraphysiological levels of peroxynitrite (due to NOS2 expression and increased ROS levels via NADPH oxidase and xanthine oxidase) are detrimental to myocardial function and decrease β-AR-stimulated cardiomyocyte contraction. This occurs via PLB dephosphorylation through PP2a activation, decreased RyR activity, mitochondrial dysfunction, and myofilament protein cleavage (Figure 2). This dual effect lends critical insight into the physiological and pathophysiological regulation of myocardial contraction by peroxynitrite.

### Conflict of interest statement

The authors declare that the research was conducted in the absence of any commercial or financial relationships that could be construed as a potential conflict of interest.
